# Digital Practices by Citizens During the COVID-19 Pandemic: Findings From an International Multisite Study

**DOI:** 10.2196/41304

**Published:** 2023-03-06

**Authors:** Hannah Ramsden Marston, Pei-Chun Ko, Vishnunarayan Girishan Prabhu, Shannon Freeman, Christopher Ross, Iryna Sharaievska, Matthew HEM Browning, Sarah Earle, Loredana Ivan, Rubal Kanozia, Halime Öztürk Çalıkoğlu, Hasan Arslan, Burcu Bilir-Koca, Paula Alexandra Silva, Sandra C Buttigieg, Franziska Großschädl, Gerhilde Schüttengruber

**Affiliations:** 1 School of Health, Wellbeing and Social Care The Open University Milton Keynes United Kingdom; 2 School of Social Sciences Monash University Melbourne Australia; 3 Systems Engineering & Engineering Management University of North Carolina Clemson, SC United States; 4 School of Nursing University of Northern British Columbia Prince George, BC Canada; 5 Department of Parks, Recreation and Tourism Management Clemson University Clemson, SC United States; 6 Communication Department National University of Political Studies and Public Administration Bucharest Romania; 7 Department of Mass Communication and Media Studies Central University of Punjab Bathinda India; 8 Department of Educational Sciences Canakkale Onsekiz Mart University Çanakkale Turkey; 9 Department of Informatics Engineering Center for Informatics and Systems at the University of Coimbra University of Coimbra Coimbra Portugal; 10 Department of Health Systems Management and Leadership University of Malta Msida Malta; 11 Institute of Nursing Science and Age and Care Research Group Medical University Graz Graz Austria

**Keywords:** COVID-19, communication, gerontology, community living, technology, social media

## Abstract

**Background:**

The COVID-19 pandemic brought digital practices and engagement to the forefront of society, which were based on behavioral changes associated with adhering to different government mandates. Further behavioral changes included transitioning from working in the office to working from home, with the use of various social media and communication platforms to maintain a level of social connectedness, especially given that many people who were living in different types of communities, such as rural, urban, and city spaces, were socially isolated from friends, family members, and community groups. Although there is a growing body of research exploring how technology is being used by people, there is limited information and insight about the digital practices employed across different age cohorts living in different physical spaces and residing in different countries.

**Objective:**

This paper presents the findings from an international multisite study exploring the impact of social media and the internet on the health and well-being of individuals in different countries during the COVID-19 pandemic.

**Methods:**

Data were collected via a series of online surveys deployed between April 4, 2020, and September 30, 2021. The age of respondents varied from 18 years to over 60 years across the 3 regions of Europe, Asia, and North America. On exploring the associations of technology use, social connectedness, and sociodemographic factors with loneliness and well-being through bivariate and multivariate analyses, significant differences were observed.

**Results:**

The levels of loneliness were higher among respondents who used social media messengers or many social media apps than among those who did not use social media messengers or used ≤1 social media app. Additionally, the levels of loneliness were higher among respondents who were not members of an online community support group than among those who were members of an online community support group. Psychological well-being was significantly lower and loneliness was significantly higher among people living in small towns and rural areas than among those living in suburban and urban communities. Younger respondents (18-29 years old), single adults, unemployed individuals, and those with lower levels of education were more likely to experience loneliness.

**Conclusions:**

From an international and interdisciplinary perspective, policymakers and stakeholders should extend and explore interventions targeting loneliness experienced by single young adults and further examine how this may vary across geographies. The study findings have implications across the fields of gerontechnology, health sciences, social sciences, media communication, computers, and information technology.

**International Registered Report Identifier (IRRID):**

RR2-10.3389/fsoc.2020.574811

## Introduction

### Background

The first cases of COVID-19 caused by SARS-CoV-2 were detected in late 2019 in Wuhan, Hubei province, China, and within months, it had spread to 113 countries in the world, leading to the declaration of a pandemic by the World Health Organization (WHO) on March 11, 2020 [1]. The COVID-19 pandemic spread across the globe and substantially impacted all aspects of daily life. Based on their cultural beliefs, political philosophies, available resources, and health care systems, nations responded differently. Several strategies, such as social distancing and isolation, case detection and contact tracing, general lockdown, and quarantine of exposed individuals, were effective in the prevention of disease spread while the virus was being studied and vaccines were being developed [1].

There is a growing body of scholarly research exploring the relationship between technology (eg, digital devices, the internet, digital gaming, social media, and mobile apps) [2,3] and loneliness [4-6]. To negotiate the constraints associated with the pandemic, technology use significantly increased, since many activities, including employment, education, health care, and other daily activities, moved to online spaces [6]. Additionally, technology has been used as a coping mechanism to follow news, get entertained, connect with others, shop online, and participate in exercise [7]. Unfortunately, despite the great range of coping strategies, loneliness prevailed among multiple groups in the population. For example, research conducted for a duration of 1 month by Groarke et al [8] at the beginning of the UK lockdown (March 23, 2020) found that the frequency of loneliness was significantly higher among younger respondents aged 18-24 years (41.0%) and 25-34 years (28.2%) than among adults aged ≥65 years (3.3%). Marital status impacted feelings of loneliness, with respondents who reported being separated or divorced (46.9%), or single or never married (40.1%) experiencing greater loneliness than those who were married/living with a partner (40.1%) or widowed (34.8%). Additionally, people who were living alone also reported higher loneliness compared to that among those with coresidents. As a result, finding ways to reduce isolation was a primary area of concern for researchers and policymakers during the pandemic, with technology use being in the forefront of this discourse as one of the potential solutions [9-18].

The pandemic brought to the fore the pivotal role the internet and Wi-Fi access played in the lives of individuals across the globe. Many individuals who conducted in-person (eg, work, leisure, and social connections) activities in the prepandemic society had to quickly transition online to ensure the same activities were achievable in this new world [7-14]. Globally, by understanding how technology was used by people living in different countries, we can better enhance our understanding of digital practices and the activities that are associated with technology use and digital practices. The United Nations (UN) [15] acknowledges that the pandemic was not only a global health crisis but also a disaster that impacted regions at the socioeconomic, security, and humanitarian levels. Further recognition notes how the pandemic has affected individuals, families, communities, and societies alike, with the UN [15] identifying strategies for socioeconomic responses.

Technology was used to reduce isolation and to address the negative outcomes of the COVID-19 pandemic and lockdowns [6,7]. The negative outcomes of the pandemic included the loss of employment and educational opportunities [5] and lack of access to the health care system [14] and mental health services, coupled with the uncertainty of the future and lack of knowledge about the virus. The issues of increased isolation and deteriorating mental health were identified as concerns during the COVID-19 pandemic [12]. To mitigate these negative outcomes, technology was employed as a possible solution [7]. This was particularly true in areas where access to technology and the internet was relatively universal [16-18]. For example, in a study conducted in April 2020 among 1374 US residents (54% female), increased use of digital communication was reported across platforms, including text messaging (43%), voice calls (36%), social media (35%), and video calls (30%) [19]. Interestingly, the same study also reported reduced digital communication use in 5% of participants during the pandemic, which included communication over social media (8%), voice calls (9%), email (10%), video calls (13%), and online gaming (17%) [19]. Younger people and women who were living alone and those who were concerned about their internet access reported increased use of digital communication, while older people reported reduced use of digital communication [19,20].

For many people globally, addressing social isolation experienced by themselves, friends, members of the community, or loved ones during lockdowns played a key role in their mental health [18,21]. Technology afforded people opportunities to remain digitally connected and explore new leisure experiences across virtual and digital environments [16,21,22]. Pennington found that social networking sites could allow users to “stay connected,” and findings from this study ascertained that respondents who were actively engaging in posts felt less loneliness than those who were engaging with individuals on a face-to-face basis [23]. Technology use to maintain contact with family and friends is common across both rural and urban environments; however, a pre–COVID-19 study exploring technology use by adults aged 70 years or older in the United Kingdom and Canada found that participants from rural communities were more positive about the use of the internet, but the viewpoint of social media platforms was negative, and these individuals did not have a social media profile and preferred to engage in face-to-face conversations [24]. Participants in rural Canada engaged with social media platforms more than participants in rural United Kingdom, and participants in the urban areas of the United Kingdom and Canada used social media and networking sites frequently [24]. These studies suggested that the experiences with technology may differ across age, geography, and other demographic characteristics. Therefore, it is important to further understand the unique differences in the relationships among technology use, social isolation, self-reported mental health and well-being, and demographic characteristics during the COVID-19 pandemic.

### Study Aims

This paper aimed to provide key insights from this exploratory descriptive study about the impact of loneliness and psychological well-being among people across different age cohorts and types of communities (eg, rural, urban, and metropolitan). Additionally, this paper will detail how technology played a role in access to community support via social media platforms from across diverse countries during the pandemic. The objectives were as follows: (1) to understand how technology played a role in access to community support for well-being; and (2) to examine the interaction among technology use, social isolation, and self-reported mental health and well-being during the COVID-19 pandemic across age, gender, home environment, and geography (including population density [rural, suburban, and urban] and country).

## Methods

### Overview

We report the methods and findings of an international multisite study conducted by a consortium of scholars from 13 countries to explore technology use, psychological well-being, COVID-19–specific questions (eg, access to support groups via social media sites), and loneliness among adults aged ≥18 years during the COVID-19 pandemic.

### Study Design

The study protocol was developed by a consortium of scholars from Austria, France, Germany, India, Malta, Portugal, Romania, Singapore, Spain, Turkey, and the United Kingdom, and has been described elsewhere [25,26]. This protocol describes the process of backward translation, the methods and approaches to participant recruitment, the different measures used in the online surveys, and the different versions of the surveys pertaining to respective legislation in countries (eg, Singapore) [25]. Two additional sites (the United States and Canada) joined the consortium after the protocol was published and therefore were not included in the earlier publication. A convenience sample was used across all countries during the rapid rollout and deployment in 2020 and 2021 [25]. A virtual snowball sampling approach was applied across the partners’ existing networks using the capabilities of the internet [27,28].

### Ethical Considerations

The study was conducted in accordance with the Declaration of Helsinki and was approved by the Human Research Ethics Committee of The Open University (protocol code HREC/3551/MARSTON). The survey was rolled out on April 4, 2020. Each partner communicated with the project lead prior to deployment of their country survey, and all respective documentation was provided to the project lead, which in turn was shared with the institutional ethics committee for an update. Data collected from this phase are referred to as Wave 1 data.

Two additional sites (the United States and Canada) joined the consortium in November/December 2020. Small changes in the wordings of the surveys were made to accommodate for differences in North American and British English, in addition to adjusting for the options available in North American communities. For example, “Ordering from a local bakery” was replaced with “Ordering take-out food,” “Streaming BBC iPlayer” was replaced with “Reading and streaming the news,” and “key worker” was replaced with “essential worker.” Additionally, response options pertaining to the question of race and ethnicity were added to follow census categories. Data collected from these 2 countries are referred to as Wave 2 data.

Informed consent was obtained from all subjects involved in the study. Each site received ethical approval: National University of Political Studies and Public Administration (SNSPA–Romania) (no protocol number; granted April 20, 2020); Open University of Catalonia (Spain); Singapore University of Social Sciences (Singapore) (no protocol number; granted April 23, 2020); Ethics Committee of the Universitat Oberta de Catalunya (Spain) (no protocol number; granted April 22, 2020); Department of Health Sciences Management and Leadership, University of Malta (Malta) (protocol number 5274_04052020; granted May 19, 2020); Department of Informatics Engineering (DEI)/Center for Informatics and Systems (CISUC) at the University of Coimbra (Portugal) (protocol number CE-057/2020_PaulaSilva; granted May 27, 2020); Department of Mass Communication and Media Studies at the Central University of Punjab (India) (protocol number CUPB/IEC/29/05/20_8; granted May 29, 2020); Nursing Science, Age and Care Research Group at the Medical University Graz (Austria); Department of Sociology at the University of Vienna, the Institute of Nursing Science at the Medical University of Graz (Austria) (protocol number 32-425 ex 19/20; June 5, 2020); the Board for the Ethical Review of Research Projects of the Institute for Communication Science (IfK) of the Westphalian-Wilhelms University of Münster (Germany) (no protocol number; granted May 7, 2020); Canakkale Onsekiz Mart University (Turkey) (protocol code 2020/83; granted June 15, 2020); Clemson University (United States) (IRB2020-435); and University of Northern British Columbia (Canada) (protocol code E2021.0323.009.00; granted May 19, 2021).

### Recruitment

Data collection for Waves 1 and 2 involved online survey invitations (deployed via Qualtrics) distributed through various professional and personal networks, mailing lists, social media platforms, snowball sampling, and the project website [28].

The Wave 1 survey (English/United Kingdom) was deployed online on April 4, 2020, and from that point onwards, consortium partners joined the project organically. The criteria for participation were as follows: (1) age of 18 years or above and (2) regular use of information and communication technology. The first wave of data was collected between April 4, 2020, and September 30, 2020, in 10 countries (Austria, France, Germany, India, Malta, Portugal, Romania, Singapore, Turkey, and the United Kingdom) and in 9 languages (Catalan, English, French, German, Hindi, Mandarin, Romanian, Spanish, and Turkish). Each survey was open for 3 months, with the English/United Kingdom survey closing on July 4, 2020. The final survey in the first wave of data closed at the end of September 2020. Wave 2 data were collected in the United States (March 29, 2021, to June 29, 2021) and Canada (June 29, 2021, to October 3, 2021).

### Materials

The survey deployed can be found in Multimedia Appendix 1 and in the study protocol [25]. The survey included multiple questions organized into several sections. Section A focused on questions relating to computer use and behavior based on previous iterations of the survey conducted in previous projects [2,3,29-31] and described in the study protocol [25]. Section B focused on COVID-19–related questions and the purpose of using technology (eg, using social media to communicate, and challenges faced). Section C focused on activities of daily living during COVID-19. These items were new and were added to the survey to capture social connections/friendships, time spent, key worker responsibilities, and giving something back [28]. Section D focused on psychological well-being [32,33] and included 18 items and 6 aspects (autonomy, environmental mastery, personal growth, positive relations with others, purpose in life, and self-acceptance of psychological well-being). The Cronbach alpha was .844. Section E focused on eHealth/digital literacy [34] and included an 8-item measure (1-5 points on a Likert scale). Section F focused on loneliness and included the UCLA Loneliness Scale version 3. This measure involves a Likert scale (1-4 points) [35], and the Cronbach alpha was .862 across all countries. It has been used to accurately measure loneliness in both younger and older populations [36-38]. This survey has been applied for wider use across the general population [35]. Section G focused on digital software technologies. These items were new and were added to the survey to capture the use of technology to relay messages via a national emergency alert system (eg, mobile app, SMS text message, etc) [2,3,39]. Section H focused on demographic questions. These included age group (18-29 years old, 30-39 years old, 40-49 years old, 50-59 years old, or ≥60 years old), gender (male, female, or prefer to self-describe), education (primary or less than high school, high school, bachelor’s degree, master’s/professional degree, or PhD), marital status (having a partner, widowed/divorced, or single), number of people staying in the same household, employment status (working, retired, or out of a job), and physical space (metropolitan/city, suburban, small town, or rural area) [2,3,24,29-31,40,41].

The study protocol [25] describes clearly and succinctly how this project was established into a multisite project. Because of national, linguist, and legal differences, there were minor changes across the different versions of the deployed surveys. This was led by each project lead (site) and the principal investigator.

### Data Analysis

Upon completion of data collection, all missing data points and data-related issues were identified and addressed. Respondents with missing data for the UCLA Loneliness Scale version 3 measure were removed prior to data analysis. Bivariate analyses were conducted to examine continuous variables (eg, UCLA 20-item Loneliness Scale version 3) among different groups based on their age, gender, type of community, etc, and a 2-sided 1-way ANOVA or Student *t* test was used based on the number of levels. For analysis of categorical variables, crosstab analyses followed by a Pearson chi-squared test and a likelihood ratio chi-squared test were performed. Ordinary least squares (OLS) regression analyses were conducted to examine to what extent the use of technology influenced the feeling of loneliness and to identify sociodemographic factors that influence the feeling of loneliness. An alpha level of *P*<.05 was used to indicate statistical significance. Estimated effect sizes were calculated with η. It should be noted that the age categories for Wave 1 and Wave 2 data were not the same because the data collected in Wave 2 included 6% of respondents aged between 50 and 59 years. To provide power for statistical tests of the data collected in Wave 2, we combined the data pertaining to respondents aged 50 years or over into a single category.

## Results

### Overview

In this section, both Wave 1 (collected in 2020) and Wave 2 results (collected in 2021) are presented. Table 1 presents a breakdown of the survey response rates.

**Table 1:** Survey response rates.

Site (country) and language		Date survey opened	Date survey closed	Sample (N=3244), n (%)
**Austria**				
	German	June 5, 2020	September 5, 2020	240 (7.4)
**Canada**				
	English	June 1, 2021	September 31, 2021	209 (6.4)
**France**				
	French	May 12, 2020	August 12, 2020	135 (4.2)
**Germany**				
	German	June 4, 2020	September 4, 2020	329 (10.1)
**India**				
	English	May 31, 2020	August 31, 2020	320 (9.9)
	Hindi	May 31, 2020	August 31, 2020	49 (1.5)
**Malta**				
	English	May 19, 2020	August 19, 2020	103 (3.2)
**Portugal**				
	Portuguese	May 29, 2020	August 29, 2020	37 (1.1)
**Romania**				
	Romanian	April 20, 2020	July 20, 2020	447 (13.8)
**Singapore**				
	English	May 17, 2020	August 17, 2020	82 (2.5)
	Mandarin	May 13, 2020	August 13, 2020	17 (0.5)
**Spain/South America**				
	Catalan/Spanish	May 4, 2020	August 4, 2020	382 (11.8)
**Turkey**				
	Turkish	June 29, 2020	September 29, 2020	108 (3.3)
**United Kingdom**				
	English	April 3, 2020	July 4, 2020	548 (16.9)
**United States**				
	English	March 29, 2021	June 18, 2021	238 (7.3)

### Lockdown Directives

Lockdown measures were implemented at different times across the different sites, starting as early as February and continuing until spring 2021 [42-61]. The measures implemented by respective national and regional governments varied considerably across the different sites [42-61], with several varying forms of directives being implemented across different states, provinces, and counties. Such measures included closure of all nonessential shops and retail outlets, introduction of education and work from home orders [42-61], enforcement of curfews (eg, 6 PM to 6 AM/9 PM to 6 AM) [49,58], enforcement of fines [49,51-57,59], enforcement of border controls [42-61], adoption of appropriate measures for people coming into the country [44-46,52,61], and requirement of documentation for proof of purpose (eg, grocery shopping/medicines, or going to work/emergency work) for leaving the home during lockdown [49,58]. In some instances, older adults (age ≥65 years) were allowed to leave their homes between 11 AM and 1 PM [59], while in other regions, roadblocks were used to monitor travel [49,58] and police were deployed onto public transport networks (eg, train services) [52-57].

### Respondent Characteristics

Table 2 presents various sociodemographic variables, in addition to the scores relating to loneliness, psychological well-being, and social media app use across Waves 1 and 2. Although the goal of this research was not to compare Wave 1 and Wave 2 data, it was observed through the data collected and analyzed that respondents in Wave 2 reported greater loneliness and experienced lower levels of psychological well-being when compared with the findings from the data collected during Wave 1. Data analysis of the UCLA Loneliness Scale version 3 measure showed that respondents did experience loneliness during Wave 1 (mean 48.11, SD 6.26) and Wave 2 (mean 49.63, SD 9.40). Psychological well-being was greater among respondents in Wave 1 (mean 69.04, SD 10.21) than among respondents in Wave 2 (mean 60.42, SD 10.73). Regarding the number of social media apps used by the respondents, most respondents across both waves used 3 to 4 social media apps, while few respondents used ≥5 social media apps.

Table 3 presents data relating to respondents who reported joining a specific online COVID-19 support group. Overall, less than 40% of the respondents reported being a member of an online community group, with a lower proportion in Wave 1 (265/1187, 22.3%) than in Wave 2 (132/337, 39.2%). From the data collected in Wave 2, the only significant association was between the use of social networking messengers (eg, Facebook Messenger, Snap Chat, etc) and the UCLA Loneliness Scale score (t_347_=3.79; *P*<.001). The levels of loneliness were higher among respondents who reported using social networking messengers than among those who did not use social networking messengers.

On investigating the impact of loneliness based on the type of community respondents reported living in, there was no significant difference in the data for Wave 1. Moreover, there was no statistical significance or interaction effects between the type of community the respondents lived in and technology use for Wave 1 data.

However, observations were ascertained and significant differences were identified from the data collected in Wave 2 (*F*_3,344_=3.28; *P*=.02). The levels of loneliness were higher among respondents living in a small town (n=115; mean 51.16, SD 8.27) than among those living in a suburban area (n=124; mean 47.82, SD 10.22; *P*=.03). There were no other significant findings involving the different types of communities and the levels of loneliness from the data collected in Wave 2.

Wave 2 data showed a significant main effect based on the feeling of loneliness and the number of social media apps used (*F*_3,332_=4.67; *P*=.003). Respondents who used no social media apps reported the lowest levels of loneliness (n=34; mean 43.88, SD 9.80). Additionally, the findings ascertained significance among respondents who were using 1 or 2 social media apps (n=116; mean 50.16, SD 8.36; *P*=.002), 3 or 4 apps (n=152; mean 50.58, SD 9.30; *P*<.001), and ≥5 apps (n=46; mean 50.85, SD 10.83; *P*=.004). There were no other significant findings involving the number of social media apps used by the respondents and the feeling of loneliness from the data collected in Wave 2. Moreover, the type of community where the respondents lived was included as an independent variable to investigate any potential interaction effects between these variables. However, data analysis showed that there were no significant interaction effects (*F*_9,332_=0.98; *P*=.45).

Wave 1 data showed that there were no differences in psychological well-being among the types of communities the respondents lived in. However, Wave 2 data showed that there was a significant main effect based on the type of community respondents lived in and their psychological well-being (*F*_3,361_=4.86; *P*=.003) (Table 4).

In Wave 2, the levels of well-being were significantly lower among respondents who reported living in a rural area (n=35; mean 53.91, SD 14.02) than among those who reported living in a small town (n=119; mean 61.37, SD 10.89; *P*=.002) or a suburban area (n=131; mean 60.75, SD 11.13; *P*=.006). Data analysis of Wave 2 showed no other significant differences involving the type of community respondents lived in and their psychological well-being. Moreover, there were no significant differences (*F*_3,349_=1.28; *P*=.28) on investigating the interaction of the type of community and the number of social media apps used with the psychological well-being of the respondents.

Tables 5 and 6 present OLS models based on the respondent characteristics and how technology use influences the feeling of loneliness according to the data collected in Waves 1 and 2, respectively. The OLS models include the independent variables of age, gender, education level, marital status, employment status, residence area, number of people living together in the same home environment, and psychological well-being, and the dependent variable of loneliness.

Two additional independent variables were included in the models and relate to the use of technology (number of social media apps used and joining a specific online COVID-19 support group). These specific independent variables were selected based on the research objective, which aimed to investigate the effects of technology use on the levels of loneliness experienced by the respondents while controlling for the characteristics during the COVID-19 pandemic. For each OLS model (Wave 1 and Wave 2), data reporting includes the estimated unstandardized coefficient (β) and standard error. Furthermore, we include the adjusted R-squared to describe the model fit.

For Wave 1, there was no association between technology use and loneliness scores. However, the levels of loneliness were higher among respondents who reported being single than among those who reported having a partner (*P*<.001). The levels of loneliness were higher among respondents who reported being unemployed than among those who reported being employed (*P*=.03). Moreover, the levels of loneliness were lower among respondents who reported having a PhD degree (*P*<.001), a master’s degree or a professional degree (*P*<.001), a bachelor’s degree (*P*<.001), or a high school level of education (*P*=.003) than among those who reported having a primary school level of education or no formal education at all. Data analyses showed that there were no differences among respondents located in European countries and the other countries.

Table 6 presents the data collected during Wave 2. The levels of loneliness were higher among respondents who reported being aged between 30 and 39 years than among those who reported being aged between 40 and 49 years (*P*=.04) or those who reported being aged ≥50 years (*P*=.01). Loneliness scores were higher among male respondents than among female respondents (*P*=.04). Furthermore, the levels of loneliness were higher among respondents who reported being unemployed or retired (*P*=.02) than among those who reported being employed (*P*=.04). Moreover, the levels of loneliness were higher among respondents who reported using one or more social media messaging apps (eg, Facebook, Snapchat, WhatsApp, etc) than among those who reported not using any social media messaging apps (*P*=.003).

Table 7 presents data related to the psychological well-being of the respondents during Waves 1 and 2. The number of social media apps used and whether respondents joined (via a social media platform such as Facebook) a specific online COVID-19 support group were statistically significant. Psychological well-being was observed to be worse among respondents who reported living in a small town than among those who reported living in a metropolitan area or a city community (coefficient=−1.974; *P*=.004).

Psychological well-being was more likely to be worse among respondents who reported being single than among those who reported having a partner (coefficient=−1.768; *P*<.05). Psychological well-being was lower among respondents aged between 18 and 29 years than among those aged between 30 and 39 years (*P*=.003) and those aged between 40 and 49 years (*P*=.04). Additionally, psychological well-being was lower among male respondents than among female respondents (*P*=.006). Moreover, psychological well-being was lower among respondents who reported being unemployed than among those who reported being employed (*P*=.04). Data analysis also identified the type of community impacted in terms of psychological well-being, and psychological well-being was higher among respondents who reported living in a small-town community than among those who reported living in a metropolitan or city community (*P*=.004).

**Table 2:** Sociodemographic characteristics.

Characteristic		Wave 1 (N=1187), n (%)	Wave 2 (N=337), n (%)
Member of a support group on social media		265 (22.3)	132 (39.2)
**Number of social messaging apps used**			
	0	43 (3.6)	31 (9.2)
	1-2	427 (36.0)	113 (33.5)
	3-4	454 (45.9)	147 (43.9)
	≥5	172 (14.5)	46 (13.4)
**Age group (years)**			
	18-29	314 (26.5)	120 (35.6)
	30-39	284 (23.9)	92 (27.3)
	40-49	303 (25.5)	64 (19.0)
	50-59	161 (13.6)	23 (6.8)
	≥60	125 (10.5)	38 (11.3)
**Gender**			
	Male	340 (28.7)	91 (27.0)
	Female	831 (70.0)	242 (71.8)
	Nonbinary	8 (0.7)	2 (0.5)
	Choose not to answer	8 (0.7)	2 (0.7)
**Education level**			
	Primary or less than high school	58 (4.9)	11 (3.3)
	High school	177 (14.9)	23 (6.8)
	College diploma/some college or university	N/A^a^	107 (31.8)
	Bachelor’s degree/professional degree	321 (27.0)	85 (25.2)
	Master’s degree	416 (35.0)	62 (18.4)
	PhD	215 (18.1)	49 (14.5)
**Marital status**			
	Having a partner/married	628 (52.9)	194 (57.6)
	Divorced/separated	82 (6.9)	12 (3.6)
	Widowed	34 (37.3)	7 (2.1)
	Single	443 (2.9)	121 (35.9)
	Prefer not to say	0 (0)	3 (0.9)
**Employment status**			
	Employed	844 (71.1)	264 (78.3)
	Retired	58 (4.9)	30 (8.9)
	Not employed (out of a job or due to other reasons)	285 (24.0)	43 (12.8)
**Type of community (residence)**			
	Metropolitan/city	608 (51.2)	74 (22.0)
	Suburban	233 (19.6)	117 (34.7)
	Small town	188 (15.8)	113 (33.5)
	Rural area	158 (13.3)	33 (9.8)
**Number of people living in the home environment**			
	1	182 (15.3)	41 (12.2)
	2	416 (35.1)	137 (40.7)
	3	222 (18.7)	58 (17.2)
	4	238 (20.1)	49 (14.5)
	≥5	129 (10.9)	52 (15.4)
**Region**			
	Europe	821 (69.2)	N/A
	North America	124 (10.5)	337 (100.0)
	Asia, Middle East, or South America	242 (20.4)	N/A

^a^N/A: not applicable.

**Table 3:** Impact of technology use on loneliness scores.

Variable		Wave 1		Wave 2	
		Loneliness score, mean (SD)	*P* value	Loneliness score, mean (SD)	*P* value
**Member of an online community support group**			.49		.29
	Yes	43.88 (5.65)		48.87 (8.86)	
	No	44.18 (6.42)		49.96 (9.96)	
**Use of the internet to stay connected with friends, family, or peers**			.50		.25
	Yes	48.46 (6.97)		49.78 (6.91)	
	No	48.27 (7.30)		48.21 (8.57)	
**Use of social media platforms**			.21		.18
	Yes	48.47 (6.97)		49.66 (9.35)	
	No	48.02 (7.44)		46.20 (11.7)	
**Use of social networking messengers**			.23		<.001
	Yes	48.41 (6.98)		50.15 (9.28)	
	No	49.47 (7.23)		43.89 (9.36)	

**Table 4:** Impact of the type of community on psychological well-being in Wave 2.

Community comparison	Mean difference	95% CI	*P* value
Metro/city vs rural	4.73	−0.96 to 10.43	.14
Metro/city vs small town	−2.71	−6.78 to 1.34	.31
Metro/city vs suburban	−2.10	−10.43 to 0.96	.53
Small town vs rural	7.45	2.05 to 12.85	.002
Suburban vs rural	6.83	1.49 to 12.18	.006
Small town vs suburban	0.62	−2.93 to 4.18	.97

**Table 5:** Wave 1 ordinary least squares regression of sociodemographic characteristics and use of technology regarding loneliness scores.

Variable			Coefficient^a^ (SE)
**Age group (reference: 18-29 years)**			
	30-39 years	0.170 (0.61)	
	40-49 years	0.722 (0.62)	
	50-59 years	0.459 (0.71)	
	≥60 years	−0.378 (0.87)	
**Gender (reference: female)**			
	Male or nonbinary/refused to answer	−0.045 (0.39)	
**Education level (reference: primary or less than high school)**			
	High school	−2.756 (0.94)^b^	
	Bachelor’s degree	−3.622 (0.90)^c^	
	Master’s/professional degree	−3.981 (0.88)^c^	
	PhD	−4.073 (0.95)^c^	
**Marital status (reference: having a partner)**			
	Divorced/separated/widowed	0.568 (0.67)	
	Single	2.441 (0.48)^c^	
**Employment status (reference: working)**			
	Retired	−0.883 (1.01)	
	Not working (out of a job or due to other reasons)	1.045 (0.49)^d^	
**Residence (reference: metropolitan/city)**			
	Rural	−0.025 (0.58)	
	Small town	0.959 (0.53)	
	Suburban	0.564 (0.48)	
**Number of people living in the home environment (reference: 1)**			
	2	0.094 (0.61)	
	3	−0.674 (0.67)	
	4	−0.368 (0.68)	
	≥5	−0.223 (0.79)	
**Number of social media apps used (reference: 0)**			
	1-2	−0.549 (0.98)	
	3-4	−0.852 (0.98)	
	≥5	−0.799 (1.06)	
**Joining a specific online COVID-19 community support group on social media (reference: no)**			
	Yes	−0.44 (0.43)	

^a^Adjusted R^2^=0.059.

^b^*P*<.01.

^c^*P*<.001.

^d^*P*<.05.

**Table 6:** Wave 2 ordinary least squares regression of sociodemographic characteristics and use of technology regarding loneliness scores.

Variable			Coefficient^a^ (SE)
**Age group (reference: 18-29 years)**			
	30-39 years	1.13 (1.52)	
	40-49 years	−2.89 (1.83)^b^	
	≥50 years	−3.67 (2.15)^b^	
**Gender (reference: female)**			
	Male	2.10 (1.24)^b^	
	Prefer not to say	0.57 (7.39)	
	Nonbinary, gender fluid	4.53 (5.60)^b^	
**Education level (reference: primary or less than high school)**			
	High school	−0.04 (3.55)	
	Bachelor’s degree	3.80 (3.13)	
	College diploma/some college	1.64 (3.09)	
	Master’s/professional degree	2.24 (3.22)	
	PhD	4.72 (3.27)	
**Marital status (reference: having a partner)**			
	Divorced/separated	1.59 (2.87)	
	Widowed	−0.44 (4.03)	
	Single	2.07 (1.53)	
	Prefer not to say	3.61 (5.99)	
**Employment status (reference: working)**			
	Retired	4.42 (2.37)^b^	
	Not working (out of a job or other reasons)	3.90 (1.65)^b^	
**Residence (reference: metropolitan/city)**			
	Rural	−1.18 (2.07)	
	Small town	0.64 (1.49)	
	Suburban	−2.90 (1.47)	
**Number of people living in the home environment (reference: 1)**			
	2	−1.84 (1.89)	
	3	−1.52 (2.08)	
	4	−1.03 (2.26)	
	≥5	−0.62 (2.29)	
**Number of social media apps used (reference: 0)**			
	1-2	5.95 (1.98)^c^	
	3-4	5.96 (1.97)^c^	
	≥5	4.97 (2.30)^b^	
**Joining a specific online COVID-19 community support group on social media (reference: no)**			
	Yes	−1.02 (1.17)	

^a^Adjusted R^2^=0.081.

^b^*P*<.05.

^c^*P*<.01.

**Table 7:** Ordinary least squares regression of sociodemographic characteristics and use of technology regarding psychological well-being.

Variable		Wave 1, coefficient^a^ (SE)	Wave 2, coefficient^b^ (SE)
**Age group (reference: 18-29 years)**			
	30-39 years	−0.72 (1.00)	4.85 (1.62)^c^
	40-49 years	−0.02 (1.03)	4.06 (1.96)^d^
	50-59 years	−1.63 (1.17)	—^e^
	≥60 years	−0.29 (1.44)	—
	≥50 years	—	4.04 (2.30)
**Gender (reference: female)**			
	Male or nonbinary/refused to answer	0.26 (0.65)	−3.67 (1.31)^c^
	Prefer not to say	—	1.67 (8.03)
	Nonbinary, gender fluid	—	−5.75 (7.47)
**Education level (reference: primary or less than high school)**			
	High school	−1.77 (1.55)	−6.17 (3.69)
	Bachelor’s degree	−2.77 (1.48)	−1.75 (3.28)
	College diploma/some college	—	−2.99 (3.20)
	Master’s/professional degree	−1.38 (1.46)	−0.66 (3.36)
	PhD	−2.20 (1.57)	−2.28 (3.43)
**Marital status (reference: having a partner)**			
	Divorced/separated/widowed	−0.28 (1.12)	−0.44 (2.91)
	Widowed	—	5.68 (3.99)
	Single	−1.77 (0.80)^d^	1.88 (1.62)
	Prefer not to say	—	2.91 (6.51)
**Employment status (reference: working)**			
	Retired	0.75 (1.68)	−1.02 (2.51)
	Not working (out of a job or due to other reasons)	−0.78 (0.82)	−3.48 (1.75)^d^
**Residence (reference: metropolitan/city)**			
	Rural	1.05 (0.96)	−3.19 (2.20)
	Small town	−1.97 (0.88)^d^	4.60 (1.57)^c^
	Suburban	−0.35 (0.80)	2.82 (1.53)
**Number of people living in the home environment (reference: 1)**			
	2	−0.29 (1.01)	3.89 (1.95)^d^
	3	1.51 (1.10)	6.55 (2.16)^c^
	4	−1.92 (1.13)	3.90 (2.34)
	≥5	−2.29 (1.31)	5.88 (2.39)^d^
**Number of social media apps used (reference: 0)**			
	1-2	0.53 (1.64)	2.56 (2.14)
	3-4	0.65 (1.64)	1.60 (2.12)
	≥5	2.2 (1.76)	3.93 (2.45)
**Joining a specific** **online COVID-19 community support group on social media (reference:** **no)**			
	Yes	−0.39 (0.72)	−7.31 (1.23)^f^

^a^Adjusted R^2^=0.021.

^b^Adjusted R^2^=0.190.

^c^*P*<.01.

^d^*P*<.05.

^e^Category not present.

^f^*P*<.001.

## Discussion

### Principal Findings

This paper explored the relationship between technology use and loneliness during the COVID-19 pandemic. We observed some associations between social network messaging but only during the second wave of data collection in 2021 from respondents in North America. Loneliness scores were higher among respondents who were using social network messaging apps than among respondents who were not using such apps. We did not observe such associations during the initial first wave of data collection in 2020 across 13 other countries. Additional findings showed that gender and age of the respondents influenced loneliness scores in both waves of data collection. In the second wave, feelings of loneliness were higher among males than females and were higher among respondents aged 30-39 years than among those in older age groups.

### Comparison With Prior Work

Our findings align with the findings of other studies that social media communication and internet usage increased loneliness during the pandemic [17,18,62-64]. Individuals who were lonely tended to use social media and the internet more, which has been associated with poorer mental health and increased prevalence of anxiety and depression. Such findings call for actions and standards from schools, workplaces, and governments regarding the use and misuse of social media and the internet. A “one-size-fits-all” approach may not benefit everyone. Exploring and identifying different options should be considered to stay (remotely) connected without a negative impact on mental health.

Similar to the findings of prior studies, we observed that being a member of community support groups or being a part of a group activity had a positive impact on loneliness [65]. Additionally, the UN [15] has outlined challenges and specificities in a bid to recover regions and impacted areas identified during the pandemic. From a socioeconomic perspective, the UN response includes the following 5-point framework [15]: (1) *ensuring all essential health services are still available and protecting health systems*; (2) *helping people cope with adversity, through social protection and basic services*; (3) *protecting jobs and supporting small and medium-sized enterprises and informal sector workers through economic response and recovery programs*; (4) *guiding the necessary surge in fiscal and financial stimulus to make macroeconomic policies work for the most vulnerable and strengthening multilateral and regional responses*; and (5) *promoting social cohesion and investing in community-led resilience and response systems.* These 5 streams are connected by a strong environmental sustainability and gender equality imperative to build back better [15].

These findings further support the observation that it is not necessarily the use of the internet or social media that influences loneliness but the involvement in online communities and activities that can influence loneliness. Building on the existing reports of the UN, WHO, and Pan American Health Organization surrounding digital (eHealth) transformation [66-69] and appropriate strategies for preparing and responding to influenza pandemics, future research may investigate the direction of this relationship and the impact of social cohesion with a view to improving community and societal resilience. Yet, we identified that single respondents and those with lesser formal education were lonelier than their counterparts across both waves of data collection. Additionally, we observed that males, younger respondents (aged 18-29 years), and unemployed respondents were comparatively lonelier. These findings align with the findings of prior studies that investigated the factors associated with loneliness during the COVID-19 pandemic [8,44,70-72], although there are exceptions pertaining to the observations associated with gender. Although the UN [15] acknowledged how industries used digital transformation throughout 2020 to enable employees to continue working from home, there are still areas that were affected, resulting in an increase in unemployment and the number of hours lost. With regard to unemployment, the UN noted that it has led to a greater impact on the health and well-being of individuals and families.

Data from the first wave of collection showed that the levels of psychological well-being were lower among respondents living in small towns than among those living in metropolitan areas. However, the data collected in the second wave showed that the levels of psychological well-being were lower among respondents living in rural areas than among those living in small towns or suburban settings. Moreover, the UN [15] has mentioned how the pandemic has revealed inequalities, resulting in many challenges for service provision and frontline staff in delivering health care specifically in urban areas. According to the UN [15], these challenges are varied and include health care access, inadequate housing, poor infrastructure (eg, transport, water, and sanitation), and employment precarity. However, the UN has mentioned that cities or metropolitan areas are perceived as “hubs of resilience and human ingenuity and this crisis has shown how city dwellers can adapt overnight to new ways of working and functioning while demonstrating extraordinary solidarity and support for one another.” Further, in the second wave data set, the levels of well-being were lower among respondents living in metropolitan areas or cities than among those living in suburban areas, and those living in small towns were lonelier than those living in suburban areas.

Collectively, these findings align with the findings of some previous studies that identified how small towns and rural areas may provide fewer options than cities for in-person activities, social engagements, health care access opportunities, and other programs that influence mental health [73-79]. Still, other studies have observed how psychological distress is higher among people living in urban areas than among those living in rural areas. It appears that the data presented in this paper align with the narrative described by the UN [15], and this situation may be explained not by residing in urban areas per se but by the lack of access to outside spaces and environmental amenities (ie, green spaces) that leads to psychological distress [80,81]. The data related to respondents residing in cities and metropolitan areas indicate a greater sense of community and resilience. Moreover, analyses of Canadian data have identified age and gender differences across varying community types, use of social media, and loneliness [82].

### Contribution and Implications

This international multicenter study was launched as a rapid response to the WHO declaring a pandemic [1] and presents a snapshot of the impact technology has on people based on the type of community they live in, their age, and their marital status during the pandemic. The consortium’s goal was to provide scholars, policymakers, educationalists, and historians (in the future) an opportunity to garner a greater understanding of societal behavior and technology use during a specific timeframe. Specifically, we aimed to enhance societal understanding of the role technology plays within and across our societies when it comes to addressing environmental aging, loneliness, and isolation, which may facilitate appropriate planning for future scenarios and crises.

### Limitations

One limitation of this work is the difficulty in presenting a cross-national perspective, given the global spread of the COVID-19 pandemic and the locations of the consortium members. The rates of survey completion varied considerably across study sites. Given the nature of survey deployment, consortium partners used their own existing mailing lists, networks, and social media platforms, and with this, the English version of the survey may have reached people who were not necessarily located in the United Kingdom but abroad instead. The design of this project only provides a snapshot of experiences shared by people who have access to technology and internet services. Future research should consider collecting more representative data.

### Conclusions

Our multisite international study showed a contrary trend whereby respondents from 2 countries in 2021, who installed more social media apps on their mobile devices, experienced greater feelings of loneliness. However, these trends did not extend equally across all countries where data were collected in 2020. Additionally, we observed how some sociodemographic factors, specifically age, gender, marital status, and type of community, were associated with loneliness and psychological well-being during the pandemic. Access and use of digital technologies, the internet, and social media have increased since the beginning of the COVID-19 pandemic within developed and developing countries. Although some increases can be attributed to trends in working and studying from home, the increased use of social media platforms and messengers has also been tied to desires to stay connected with friends, families, and peers to avoid loneliness. Future studies and discourse should start to consider the role and access of transgenerational technology [83] to explore and understand the sociodemographic factors when implementing programs and activities to reduce loneliness and improve well-being across the rural and urban spectrum.

## Appendix

Multimedia Appendix 1 Online survey in Wave 1.

## Abbreviations

OLS: ordinary least squares
UN: United Nations
WHO: World Health Organization
