# Supplementary material for: Digital Practices by Citizens During the COVID-19 Pandemic: Findings From an International Multisite Study
Source: JMIR Ment Health. 2023 Mar 6;10:e41304. doi: 10.2196/41304 (PMC9994468; doi:10.2196/41304)
Supplement: Multimedia Appendix 1 [file mental_v10i1e41304_app1.pdf]

COVID-19: Technology, Social Connections, Loneliness & Leisure Activities: Wave 1 (online survey)

Question #	Item	Answer (options)	Additional information
2	Do you own a computer	Yes<br>No	
3	How long have you used a computer?	More than 15 years<br>More than 10 years<br>More than 5 years<br>For 1 year or less<br>Less than 1 year	
4	What do you use a computer for? Select all that apply	Connecting with COVID-19-support group(s)<br>Connecting with Community Groups<br>Ordering fruit/veg boxes from a local supplier.<br>Connecting with friends<br>Connecting with family<br>Ordering from a local bakery<br>Connecting with healthcare professionals<br>Writing a COVID-19 blog<br>Watching porn<br>Word processing<br>Email<br>Playing games<br>Online banking<br>Job searching<br>Accessing social media<br>Online shopping<br>For work purposes<br>For tracking/recording my data (finances/health etc.)<br>Listening to music<br>Watching TV<br>Searching the Internet<br>Paying bills<br>Other	
5	In a week, how frequently do you use a computer?	Daily<br>2-3 times a week<br>Once a week<br>Never	
6	Do you have the Internet at home?	Yes<br>No	
7	How do you usually access the Internet? Select all that apply.	via a Smartphone<br>via a PC/Laptop<br>via a Tablet<br>via the TV<br>via multiple devices	
8	How frequently do you use the Internet?	More than once a day<br>About once a day<br>More than once a week<br>Every other day<br>More than once a month	

9	What do you use the Internet for? Select all that apply.	Job searching<br>Searching for Health Information<br>Using Facebook<br>Using Twitter<br>Streaming Netflix<br>Streaming BBC iPlayer<br>Streaming other<br>Downloading activity<br>Playing games<br>Listening to music<br>Watching porn<br>Reading<br>I use the Internet for work<br>Online Banking<br>Emailing<br>Online Shopping<br>Searching for Information (e.g., weather, fact checking etc.)<br>Dating sites/apps<br>Booking travel arrangements<br>Booking holidays<br>Booking events (e.g., music events)<br>Accessing gambling sites<br>Other	
10	Do you use social networking sites (e.g. Facebook, Twitter, Snapchat, Instagram, Pinterest, LinkedIn)	Yes<br>No	
11	Which social media & communication platforms do you use? Select all that apply. Which social media & communication platforms do you use? Select all that apply.	Facebook<br>Twitter<br>Instagram<br>Snapchat<br>LinkedIn<br>What's App<br>Viber<br>Facebook Messenger<br>FaceTime<br>Pinterest<br>Skype (incl. Business)<br>Zoom<br>Telegram<br>WeChat<br>Other	
12	How long have you been using social	10-15 years<br>5-10 years<br>Less than 5 years	

	networking sites (Facebook, Twitter, Instagram, Pinterest, LinkedIn)?	Less than 1 year	
13	How frequently do you use social networking sites?	Daily<br>4-6 times a week<br>2-3 times a week<br>Once a week<br>Never	
14	Which social media platform do you use the most?	Open ended answer	
15	Which communication platforms do you use the most? Select all that apply.	What's App<br>Facebook messenger<br>Telegram<br>Viber<br>SMS<br>Telephone call<br>Snapchat<br>Facetime<br>Zoom<br>Other	
16	Why do you use social media sites? Select all that apply.	To share Information<br>To stay connected to friends<br>To stay connected with family<br>To share photographs<br>To organise events<br>To take part/follow events & groups that I am interested in<br>To keep up to date with the latest news<br>To express my opinions and views<br>To keep up to date with COVID-19 information<br>To share information about COVID-19<br>To search for local delivery companies (e.g., fruit & veg) during COVID-19<br>To keep up to date with support groups during COVID-19<br>To contact my neighbours/community for help/assistance during COVID-19<br>To contact/receive support from healthcare professionals during COVID-19<br>Other	
17	Since COVID-19 have you become a member of a community support group on social media (e.g., Facebook or similar)?	Yes<br>No	

18	Since COVID-19 are you familiar with any community support groups on social media (e.g., Facebook or similar) platforms?	Yes<br>Unsure<br>No	
19	Since COVID-19 have you had to request assistance from a loved one or the community for support (e.g., delivering groceries, prescriptions etc.)?	Yes<br>No	
20	Since COVID-19 have you signed up to receive community deliveries/assistance (e.g., groceries from a local newsagents /community shop / butcher's / farmer's market)?	Yes<br>No	
21	Since COVID-19 would you sign up to receive community deliveries/assistance (e.g., groceries from a local newsagents/community shop/ butcher's / farmer's market)?	Yes<br>Unsure<br>No	
22	Please describe why you WOULD sign up to receive community deliveries and/or assistance. If you choose not to	Open ended answer	

	answer please write N/A		
23	Please describe why you are unsure or you WOULD NOT sign up to receive community deliveries and/or assistance. If you choose not to answer please write N/A	Open ended answer	
24	Since COVID-19 how have you been spending your time? Select all that apply	• I am a key worker.<br>• Home schooling my children<br>• Communicating with specific COVID-19 support group(s) for my area.<br>• Sleeping<br>• Attending virtual exercise sessions (e.g., Yoga/Pilates/ Joe Wicks)<br>• Attending virtual community meetings (e.g., WI, Men's Sheds or similar)<br>• Attending virtual educational classes<br>• Attending work-related meetings on communication tools<br>• Working from home<br>• Self-isolating due to vulnerable loved ones<br>• Catching up reading books<br>• Watching streaming services<br>• Sharing information on social media sites<br>• Gardening/allotment<br>• Searching for activities for my child/children<br>• Connecting with friends/family virtually<br>• Online grocery shopping<br>• Virtually connecting with community support groups<br>• I am not complying (please describe your activities)<br>• Other	
25	Since COVID-19, please rate your level of concerns (relating to yourself) about the various challenges faced in society	• My death & that of people close to me (e.g., family members)<br>• Limited physical contact with loved ones<br>• Limited social contact with friends and family<br>• Food shortage<br>• Being able to pay bills (incl. rent/mortgage)<br>• My mental health<br>• The mental health of my children & family members<br>• Not having a job to go back to Eating healthy because of food allergies, and overall health.<br>• I am on my own. I have no support (e.g., no children etc.)	5-pt Likert [Strongly disagree – Strongly agree] User engages/answers via a slider [strongly disagree 0-100 strongly disagree]

		• Businesses going bust.<br>• Entering a recession<br>• Not completing my education (e.g., college/university)<br>• Education for my child/children<br>• Staying alive (due to pre-existing health conditions)	
26	What type of digital devices do you own in your home? Select all that apply	Smart phone<br>Mobile phone<br>Tablet<br>Desktop PC/Laptop	
27	Which digital device do you use most frequently? (Select 1 answer)	Smart phone<br>Mobile phone<br>Tablet<br>Desktop PC/Laptop	
28	On your digital device do you share information about COVID-19?	Yes<br>Sometimes<br>No	
29	How frequently do you share information?	Daily<br>4-6 times a week<br>2-3 times a week<br>Once a week<br>Never	
30	Why do you share information surrounding COVID-19? Select all that apply	• Because I want to update my friends and family<br>• Because it is important to share information during this pandemic<br>• Because it is my responsibility as a citizen to share information to help other citizens?<br>• Because I like to share information<br>• To express my views and opinions<br>• To stay connected with friends.<br>• To stay connected with grand/children.<br>• To stay connected with the Community support groups	
31	Do you have any concerns about sharing information surrounding COVID-19?	Yes<br>Maybe<br>No	
32	You answered YES/MAYBE to the previous question. Please describe why. If you choose not to answer please write N/A	Open ended answer	

33	You answered NO to the previous question. Please describe why. If you choose not to answer please write N/A	Open ended answer	
34	Have you changed your use & behaviour of technology during COVID-19 crisis?	Yes<br>A little<br>No	
35	How has your use & behaviour of Technology changed during COVID-19? Please describe below. If you choose not to answer please write N/A	Open ended answer	
36	During COVID-19 new experiences are happening. Please select which best suits you	• Making new social connections/friendships<br>• Sharing my knowledge & skills with the community<br>• Learning new information<br>• Learning new skills<br>• Spending more time with spouse/family members<br>• Changing my routine<br>• Sharing information across the community to help those who could be vulnerable.<br>• Being a key worker & helping in this pandemic<br>• Home schooling my children<br>• Being able to cook for my family.<br>• Being isolated more<br>• Giving something back	7-pt Likert [Strongly Agree-Strongly Disagree]
37	During COVID-19, have you volunteered to help your community?	Yes<br>No<br>Not, but I would like to	
38	Please describe how you are volunteering or would like like to get involved? If you choose not to	Open ended answer	

	answer please write N/A		
39	Since COVID-19, has the way you use your digital devices changed your way of communication?	Yes<br>A little<br>No	
40	Please describe how your communication behaviour(s) has changed since COVID-19. If you choose not to answer please write N/A.	Open ended answer	
41	Select one response against each statement to indicate how much you agree or disagree	• "I like most parts of my personality."<br>• "When I look at the story of my life, I am pleased with how things have turned out so far"<br>• "Some people wander aimlessly through life, but I am not one of them."<br>• "The demands of everyday life often get me down."<br>• "In many ways I feel disappointed about my achievements in life."<br>• "Maintaining close relationships has been difficult and frustrating for me."<br>• "I live life one day at a time and don't really think about the future."<br>• "In general, I feel I am in charge of the situation in which I live."<br>• "I am good at managing the responsibilities of daily life."<br>• "I sometimes feel as if I've done all there is to do in life."<br>• "For me, life has been a continuous process of learning, changing, and growth."<br>• "I think it is important to have new experiences that challenge how I think about myself and the world."<br>• "People would describe me as a giving person, willing to share my time with others."<br>• "I gave up trying to make big improvements or changes in my life a long time ago."<br>• "I tend to be influenced by people with strong opinions"<br>• "I have not experienced many warm and trusting relationships with others."	7-pt Likert [Strongly Agree- Strongly Disagree]

		• “I have confidence in my own opinions, even if they are different from the way most other people think.”<br>• “I judge myself by what I think is important, not by the values of what others think is important."	
42	Select one response against each statement to indicate how much you agree or disagree	• I know how to find helpful health resources on the Internet.<br>• I know how to use the Internet to answer my health questions.<br>• I know what health resources are available on the Internet.<br>• I know where to find helpful health resources on the Internet.<br>• I know how to use the health information I find on the Internet to help me.<br>• I have the skills I need to evaluate the health resources I find on the Internet.<br>• I can tell high quality from low quality health resources on the Internet.<br>• I feel confident in using information from the Internet to make health decisions.	5-pt Likert [Strongly agree-Strongly disagree]
43	Select one response against each statement to indicate how your feelings	• How often do you feel that you are "in tune" with the people around you?<br>• How often do you feel that you lack companionship?<br>• How often do you feel that there is no one you can turn to?<br>• How often do you feel alone?<br>• How often do you feel part of the a group of friends?<br>• How often do you feel that you have a lot in common with the people around you?<br>• How often do you feel that you are no longer close to anyone?<br>• How often do you feel that your interests & ideas are not shared by those around you?<br>• How often do you feel outgoing & friendly?<br>• How often do you feel close to people?<br>• How often do you feel left out?<br>• How often do you feel that your relationships with other are not meaningful?<br>• How often do you feel that no one really knows you well?<br>• How often do you feel that there are people who really understand you?<br>• How often do you feel shy?<br>• How often do you feel that people are around you but not with you?<br>• How often do you feel that there are people you can talk to?	4-pt Likert [Never-Often]

		• How often do you feel that are people you can turn to?<br>• How often do you feel isolated from others?<br>• How often do you feel you can find companionship when you want it?	
44	Since COVID-19, has your communication changed within your social networks?	Yes<br>A little<br>No	
45	During the COVID-19 Pandemic how frequently have you communicated with members of your social network? Please rate your answers below on each of the sliders	Friends<br>Work colleagues<br>Members from a community group (e.g., Women's Institute, Church, Men's Sheds etc.)<br>COVID-19 Community Support Group<br>Family members<br>Children<br>Grandchildren	User engages/answers via a slider [Not at all 0-100 Very Frequently]
46	Does your country have a National Emergency Alert System (e.g., mobile app or SMS)?	Yes<br>Unsure<br>No	
47	Please describe what your thoughts are relating to a National Emergency Alert System (e.g., mobile app or SMS)? If you choose not to answer please write N/A.	Open ended answer	
48	Virtual Assistants such as Alexa, Google Home, Siri have various features. Please describe whether you think such devices or similar would be suitable	Open ended answer	

	as a way of sharing National Emergency Alert Systems. If you choose not to answer, please write N/A		
49	Please add any additional information that you would like to share about your experiences & day-to-day living during COVID-19 crisis. If you choose not to answer, please write N/A	Open ended answer	
50	What type of community do you live in?		
51	What is your age?	18-95+ years<br>Split up into interval categories (e.g., 18-24, 25-29)	
52	What is your highest level of education? - please select ONE answer	Less than high school degree<br>High school (E.g., O Levels, GED, GCSE)<br>Some college but no degree (E.g., BTEC, NVQ, A-Levels)<br>Bachelor's degree (3-year)<br>Master's degree<br>Doctoral degree<br>Professional degree (JD, MD)<br>Other	
53	Which best describes your ethnicity?	White (Caucasian, European, Irish)<br>Any other White background<br>Irish Traveller<br>Black or African American /British<br>Caribbean<br>Native American/ American Indian / Alaska Native<br>Pacific Islander/ Native Hawaiian<br>Hispanic/Latino (Mexican, Puerto Rican Cuban)<br>Asian British or Asian American (Indian, Bangladeshi, Pakistani, Sikh)<br>Chinese/Filipino, Vietnamese/Thai British or American<br>Chinese/Filipino, Vietnamese/Thai<br>Other Asian origin group	
54	How would you describe your gender?	Male (identify as male)<br>Female (identify as female)<br>Prefer to self describe as (non-binary, gender fluid, agender, please specify)<br>Prefer not to say	
55	Do you identify as transgender or	Yes<br>No	

	have a transgender history?	Prefer not to say	
56	Please select the sexual orientation category that best represents you:	Heterosexual (straight)<br>Gay man<br>Gay Woman / Lesbian<br>Bisexual<br>Pansexual<br>Prefer not to say<br>Prefer to self-describe	
57	Marital Status	Single<br>Married<br>Widowed<br>Divorced<br>Separated<br>Civil Partnership<br>Prefer not to say	
58	How many people (including yourself) are living or staying at your address? (please select 1 answer)	1<br>2<br>3<br>4<br>5<br>6<br>More than 6	
59	How many people include children (under 18 years old) are living at your address? (please select 1 answer)	0<br>1<br>2<br>3<br>4<br>5<br>6<br>More than 6	
60	Do you meet the criteria for being vulnerable or extremely vulnerable?	Yes<br>No	
61	Which statement best describes your current employment status?	Working (paid employee)<br>Working (self-employed)<br>Not working (furlough because of COVID-19)<br>Not working (looking for work)<br>Not working (retired)<br>Not working (disabled)<br>Not working (other)	
62	Which of the following industries most closely matches your employment/	Education (e.g., Academic/Researcher/Doctoral Student)<br>Entertainment/Tech Industry<br>Self-employed<br>I am a student (e.g., part time, full time)<br>Forestry, fishing, hunting or agriculture support<br>Real estate or rental and leasing<br>Homemaker	

	past employment?	Professional, scientific or technical services<br>Utilities<br>Management of companies or enterprises<br>Admin, support, waste management or remediation services<br>Manufacturing<br>Educational services<br>Wholesale trade<br>Health care or social assistance<br>Retail trade<br>Arts, entertainment or recreation<br>Transportation or warehousing<br>Accommodation or food services<br>Information<br>Other services (except public administration)<br>Finance or insurance<br>Other	
63	Do you consider yourself to have a disability?	Yes<br>No<br>Prefer not to say	
64	In which country do you currently reside?	Drop down menu for selection	
65	Have you had to self-isolate because of COVID-19?	Yes<br>No	
